# The Effect of Efavirenz on Estradiol Metabolism in Transgender Women

**DOI:** 10.1089/trgh.2019.0018

**Published:** 2019-09-04

**Authors:** Matthew C. Leinung, Cynthia H. Miller, Babak Tehrani, Jalaja Joseph

**Affiliations:** ^1^ Division of Endocrinology, Albany Medical College, Albany, New York.; ^2^ Division of HIV Medicine, Albany Medical College, Albany, New York.; ^3^ Department of Medicine, Albany Medical College, Albany, New York.

**Keywords:** efavirenz, estradiol, HIV, medroxyprogesterone, metabolism, transgender

## Abstract

The goal of hormonal therapy in treating gender dysphoria is to maintain cross-sex hormone levels in the normal physiological range for the desired gender. Estrogen is the mainstay of hormonal therapy for male to female transgender patients. Efavirenz, a non-nucleoside reverse-transcriptase inhibitor, has been one of the most commonly prescribed antiretroviral therapies (ARTs). However, this regimen has also given rise to the most clinically significant drug–drug interactions between ARTs and hormone-based contraceptives. We discuss here three transgender HIV-positive women in whom efavirenz effected the metabolism of orally administered estradiol (and probably medroxyprogesterone).

## Introduction

Transgender individuals have a gender identity that is discordant with the gender assigned to them at birth. Increasing numbers of these individuals are seeking hormonal therapy to align their physical appearance with their perceived gender.^1^ Estrogen is the mainstay of hormonal therapy for male to female transgender patients. The goal is to maintain cross-sex hormone levels in the normal physiological range for the desired gender.^2^ In most patients, oral delivery of estradiol (up to 6 mg daily) is able to achieve normal female 17-β estradiol levels and suppression of testosterone, thus enabling physical changes concordant with female phenotype.^3,4^

Efavirenz, a non-nucleoside reverse-transcriptase inhibitor, has been one of the most commonly prescribed antiretroviral therapies (ARTs). Although efavirenz-based ART has been a preferred first-line regimen for treatment-naive patients, this regimen has also given rise to the most clinically significant drug–drug interactions between ARTs and hormone-based contraceptives. Although data are limited and inconsistent, it has been recommended that oral contraceptives not be relied upon to prevent pregnancy when taking efavirenz.^5^ Although efavirenz is no longer a first-line therapy in HIV care in the Western world, it is still used frequently elsewhere due to cost and other considerations. Hence, drug–drug interactions with efavirenz can be clinically important.

We discuss here three transgender HIV-positive women on estradiol therapy and how the serum 17-β estradiol levels were affected by concomitant use of efavirenz.

## Materials and Methods

The Transgender Clinic at Albany Medical Center has provided hormonal therapy for >400 transgender individuals over the past 2 decades, including 22 individuals who are HIV positive. For trans women, we typically use oral estradiol in doses of 4–6 mg daily. The goal range of serum 17-β estradiol is 100–200 pg/mL.^2,3^ If this level is not achieved (∼15%), we consider increasing to 8 mg daily. If serum testosterone is not well suppressed (<100 ng/dL) despite full dose estrogen therapy (occurring in ∼25%), we may add medroxyprogesterone acetate (MPA) for its gonadal suppressing effects. We often provide antiandrogen therapy with either spironolactone or finasteride (∼70% of patients). All therapies are individualized, based upon measured hormone levels, comorbidities, and consideration of patient preferences.

Total testosterone in our clinic is determined by chemiluminescent immunoassay (Access Testosterone Assay; Beckman Coulter, Inc., CA). Our internal reference ranges for this assay are as follows: normal male >320 ng/dL; equivocal 200–319 ng/dL; hypogonadal <200 ng/dL.^3^ The female normal reference range was set at 10–75 ng/dL. Serum 17-β estradiol levels are determined by chemiluminescent microparticle immunoassay (Architect Estradiol assay; Abbott Laboratories, IL). The normal male reference range is 11–44 pg/mL, and normal menstruating female follicular phase reference range is 21–251 pg/mL. The Clinical Chemistry Lab at Albany Medical Center is certified for estradiol testing by New York State Department of Health that accepts CAP (College of American Pathology) proficiency as an index of performance.

*Patient 1* is a 29-year-old HIV-positive trans woman who, on 6 and 8 mg orally of estradiol, had 17-β estradiol levels <50 pg/mL on multiple determinations with no suppression of serum testosterone levels (values >300 ng/dL). MPA 5 mg daily was added to no effect. Her HIV medication regimen included Atripla (efavirenz 600 mg/day, tenofovir disaproxil fumarate 300 mg daily, and emtricitabine 200 mg daily), which was then switched to Odefsey (rilpivirine, tenofovir alafenamide, and emtricitabine). She also requested a change at this time from estradiol to conjugated estrogen (Premarin), while remaining on MPA. On this regimen, her testosterone level dropped to 73 ng/mL (serum 17-β estradiol not measured as she is on conjugated estrogen).^6^

*Patient 2* is a 56-year-old trans woman who was found upon repeated determinations dating back to 2011 to have poor suppression of serum testosterone (values 127–298 ng/dL) and minimal elevation of serum 17-β estradiol levels (ranging from undetectable to 56 pg/mL) despite receiving daily 8 mg oral estradiol, 200 mg spironolactone, and 10 mg oral MPA. Her HIV regimen was Atripla once daily.

In December 2015, she underwent vaginoplasty and orchiectomy with a decrease in serum testosterone levels to <30 ng/mL, but her serum 17-β estradiol level remained low (45 pg/mL) on 8 mg estradiol (MPA was discontinued as testosterone suppression was no longer needed). She was then switched to estrogen patch, delivering 0.1 mg/24 h, with a rise in her 17-β estradiol level to 148 pg/mL.

*Patient 3* is a 52-year-old HIV-positive trans woman on an estradiol patch, 0.05 mg/day, with a serum 17-β estradiol level of 54 pg/mL. Her HIV regimen includes Sustiva (efavirenz). She has previously undergone orchiectomy.

## Results

The low serum 17-β estradiol levels in patients 1 and 2 were unusual compared with those in >150 patients seen in our clinic (where the mean is >100 pg/mL on doses of 6 or 8 mg daily).^3^ Oral estradiol dosing and serum hormone levels in our HIV-positive transgender female patients not receiving efavirenz are not different compared with those in HIV-negative patients (data not shown).^3^ Given the change in the serum 17-β estradiol level seen in patient 2 after moving to the transdermal route, we then looked at 17-β estradiol levels in transgender women treated with an estrogen patch (*n*=15). On transdermal therapy delivering 0.05 mg estradiol daily, the mean serum 17-β estradiol level is 34.9 pg/mL, whereas on a patch delivering 0.10 mg daily, the mean serum 17-β estradiol is 91.8 pg/mL. We observed that patient 3, on efavirenz and a patch delivering 0.05 mg/daily, had a serum 17-β estradiol level of 54 pg/mL, which is comparable with other transgender women on that dose.

## Discussion

Maintaining cross-sex hormone levels in the normal physiological range of the desired gender is the goal of hormonal therapy for transgender patients. For transgender females, this is typically done with administration of oral estradiol in doses of 4–6 mg daily. Although there is wide variability between individuals, two recent reports have shown the effectiveness of this approach for most patients, as serum 17-β estradiol levels rise into the average adult female range and serum testosterone levels suppress well below the normal adult male range.^3,4^

We describe here two patients receiving efavirenz-based ART in whom we were unable to obtain the expected serum 17-β estradiol levels and testosterone suppression with high-dose oral estradiol and MPA. In patient 1, a previously nonsuppressible testosterone level dropped significantly after moving away from efavirenz-based ART. In patient 2, a goal level of serum 17-β estradiol was achieved only after changing the method of delivery to the transdermal route. In addition, patient 3 on efavirenz and a low transdermal dose of estradiol was noted to have serum 17-β estradiol levels comparable with those of other trans women receiving the same estrogen regimen. We reviewed the response to oral estradiol in other HIV-positive patients not on efavirenz and did not encounter an inability to achieve desired serum estradiol levels.

Although none of these cases alone is sufficient to make a compelling case, these results suggest to us that efavirenz affects the metabolism of oral estradiol (and probably MPA). Patient 1 requested a change to a conjugated estrogen formulation while we changed her ART, thus preventing us from reliably measuring her serum 17-β estradiol level.^6^ We gave her a comparable dose (5 mg) of Premarin and continued MPA, which was accompanied by suppression of her serum testosterone. In patient 2, the switch to the transdermal route gave her, for the first time, a serum estradiol level in the goal range. We could not see what effect this had on her testosterone level as she had, by that time, undergone orchiectomy. Patient 3 was treated with a low dose of transdermal estradiol due to a history of a thrombotic stroke while abusing street drugs. Before assessing the response of her testosterone level to the transdermal estradiol, she underwent orchiectomy.

This interaction of efavirenz and contraceptives has been noted previously in articles describing decreased effectiveness of oral contraceptives with concomitant use of efavirenz.^5^ This may be due to the ability of efavirenz to stimulate CYP3A4, which is involved in estradiol and progestin metabolism.^7^ Transdermal administration may bypass this first-pass liver effect. A recent review of the effects of efavirenz concluded that depot MPA was not affected whereas oral contraceptives and perhaps implants lost effectiveness. Conversely, a recent report found that the implantable route did not seem to prevent accelerated metabolism and contraceptive effectiveness of the progestin levonorgestrel.^8^ These discordant results may reflect the uncertainty of the minimal effective dose for contraception and the degree of first pass liver metabolism.

## Conclusions

In summary, we found evidence that efavirenz effects the metabolism of oral estradiol (and probably MPA) in transgender women. We recommend hormone levels be used to help guide the individualization of therapy when treating transgender women receiving efavirenz-based ART.

## Authorship

All authors have read and approved the final article. M.L., C.M., and J.J. wrote the article. M.L. and B.T. collected and analyzed the data.

## Ethics Statement

All procedures performed in studies involving human participants were in accordance with the ethical standards of the institutional and/or national research committee and with the 1964 Helsinki declaration and its later amendments or comparable ethical standards.

This report was done as part of a longitudinal study of our transgender population that was approved by our internal IRB. Informed consent was not deemed necessary, as the data were observational, with no experimental intervention, and patient confidentiality was maintained.

## Abbreviations Used

ART: antiretroviral therapy
MPA: medroxyprogesterone acetate
